# Multiple invasions and predation: The impact of the crayfish *Cherax quadricarinatus* on invasive and native snails

**DOI:** 10.1002/ece3.11191

**Published:** 2024-04-01

**Authors:** Thomas Baudry, Lola Millet, Philippe Jarne, Patrice David, Frédéric Grandjean

**Affiliations:** ^1^ Laboratoire Ecologie et Biologie Des Interactions, UMR CNRS 7267 Equipe Ecologie Evolution Symbiose Université de Poitiers Poitiers Cedex France; ^2^ CEFE, CNRS ‐ Univ Montpellier ‐ IRD – EPHE Montpellier Cedex 5 France

**Keywords:** chemical stimuli, escape behavior, mollusks, prey–predator interactions, refuge size, survival

## Abstract

The pace of biological invasions has increased in recent decades, leading to multiple invasions and the potential dominance of invasive species, destabilizing local ecological networks. This provides opportunities to study new ecological species interactions, including predation. Tropical freshwaters have been particularly concerned by aquatic invasions and we focused here on the Martinique island (Lesser Antilles). We examined the predator–prey relationships involving invasive Thiarid snails (*Tarebia granifera* and *Melanoides tuberculata*) and the native *Neritina punctulata*, both confronted with a newcomer predator, the redclaw crayfish (*Cherax quadricarinatus*). We conducted several mesocosm experiments to assess the impact of crayfish predation on snail survival and the passive and active antipredator responses of snails. A first experiment indicated snail survival rates between 50% and 80%, depending on crayfish size and sex. Notably, there was a negative correlation between snail survival and male crayfish size and the predation method (shell crushing vs. “body sucking”) varied with crayfish size. The second experiment suggested no refuge size for snails, with both very small (<5 mm) and very large (>5 mm) unable to escape predation, regardless of crayfish size (from 77 to 138 mm) or sex. Finally, we investigated the escape behavior of Thiarids regarding three crayfish cues. *Melanoides tuberculata* tend to bury in the substrate and *T. granifera* to climb up aquarium walls, what was expected from their shell morphologies, and both responding to crayfish cues within minutes. Overall, *C. quadricarinatus* proves to be an efficient snail predator with limited escape options for snails, potentially contributing to the decline of certain snail populations in Martinique. This omnivorous predator might impact other native species across different groups, including shrimps and fish. Our study underscores the urgent need for monitoring efforts, solidifying the redclaw crayfish reputation as a dangerous invasive species for freshwater macrobenthic faunas worldwide.

## Graphical abstract

We investigated predator–prey interactions involving Thiarid snails that invaded Martinique several decades ago and the recently introduced predator, the redclaw crayfish (*Cherax quadricarinatus*), unveiling several critical insights: (1) Crayfish predation impact on snail survival: Our experiments indicate snail survival rates ranging from 50% to 80%, depending on crayfish size and sex. The negative correlation between snail survival and male crayfish size, as well as the variation in predation methods, represents a key aspect of the predator–prey dynamics. (2) Lack of refuges for snails: Our study demonstrates that both very small (<5 mm) and very large (>5 mm) snails are unable to escape predation, irrespective of crayfish size or sex. This observation underscores the formidable efficiency of the redclaw crayfish as a predator. (3) Snail escape behavior in response to crayfish cues: We also shed light on the distinct responses of two snail species to cues emitted by crayfish, revealing notable differences in their escape behaviors.

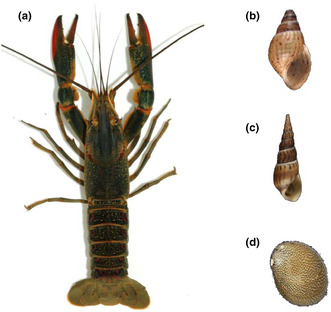

## 1 INTRODUCTION

The stability of biological communities is often threatened by Invasive Alien Species (IAS) because local species have not coevolved with them (Chase, 2003; Leibold & Chase, 2018). The impact of introduced predators in particular is often extremely negative, because prey may have developed defenses against local predators, but not against introduced ones. These defenses include predator recognition through various signals (e.g., chemical), behavioral escape, and morphological reinforcement (Alexander & Covich, 1991; Krist, 2002; Vermeij, 1978). Introduced generalist, high‐rank predators often have the strongest impacts because they affect many local species at a time, potentially destabilizing whole ecosystems. An iconic example is the Nile perch which strongly depressed the dynamics of numerous cichlid species in large African lakes (Aloo et al., 2017; Lowe et al., 2000). Several crayfish species are also among the invaders with strong impacts, able to feed on mollusks (Haubrock et al., 2021; Herrmann et al., 2022; Sanders & Mills, 2022), other benthic invertebrates (shrimps, insect larvae; Ercoli et al., 2015; Mathers et al., 2020; Twardochleb et al., 2013), and macrophytes (Gherardi & Acquistapace, 2007; Souty‐Grosset et al., 2016).

Biological invasions have become so frequent that it is not uncommon to witness colonization by successive IAS, a process sometimes called overinvasion (Dubreuil et al., 2021; Firmat et al., 2013; Russell et al., 2014). Overinvasions have been widely documented both at large geographic scales leading to fauna homogenization (e.g., Soto et al., 2023), or in more local situations (Dubreuil et al., 2021; James et al., 2016; Linzmaier et al., 2018). They may lead to predation by IAS on other IAS, potentially controlling their dynamics or changing their relative abundance in the community. To predict this impact, one must document the IAS predation efficiency in its new environment (Chabrerie et al., 2019; Mack, 2003), and the characteristics of the different IAS prey, that make them more or less sensitive to predation, including morphological and behavioral resistance traits (Chabrerie et al., 2019; Wisz et al., 2013), or even the habituation to predator presence (Holomuzki & Hatchett, 1994; Sih et al., 2010).

Aquatic ecosystems have been particularly impacted by IAS (Cuthbert et al., 2021; Gallardo et al., 2016), often brought by aquarium trade or aquaculture (Gherardi et al., 2008; Nunes et al., 2015; Rodríguez‐Barreras et al., 2020). Freshwaters of tropical islands are particularly vulnerable, characterized by low species diversity combined to high endemism, absence of top predators, and therefore simple food webs (Nivet et al., 2010) on small areas (Myers et al., 2000). IAS may end up dominating these communities, as for example in Puerto Rico (*ca.* freshwater fish species identified with only 20% being native; Rodríguez‐Barreras et al., 2020). This is also true of Martinique (Lesser Antilles): its original freshwater fauna of fish, crustaceans, and snails did not exceed 40–50 species, and it now counts *ca.* twice as many (Delannoye et al., 2015; Lim et al., 2002; authors' data). In particular, two snail species of the family Thiaridae (*Melanoides tuberculata* and *Tarebia granifera*) have been extremely efficient IAS. Introduced in the late 1970s and early 1990s, respectively, as a consequence of aquarium trade, these two species now occupy most of Martinique hydrographic network, competing against each other and with local species (Delannoye et al., 2015; Dubart et al., 2019; Facon et al., 2008), and making up most of the benthic animal biomass in Martinican rivers (Dubart, 2019; Facon et al., 2008; Facon & David, 2006).

A newcomer, the redclaw crayfish *Cherax quadricarinatus*, could however change the game. This species represents a new threat to freshwater ecosystems in the tropics worldwide (Haubrock et al., 2021). Introduced in Martinique for aquaculture purpose in the early 2000s, it has colonized the major watersheds in a decade through human releases, with densities of up to five individuals per m^2^, and is characterized by a very large adult size (up to 23 cm; Baudry et al., 2020, 2021). Its impact on local communities has not been studied, but its spread temporally coincides with an apparent decline of Thiarid populations in some rivers (P. Jarne & P. David, unpublished data). Crayfish are indeed known as snail predators (Herrmann et al., 2022; Hofkin et al., 1992; Souty‐Grosset et al., 2016), sometimes strongly driving down local populations (e.g., the crayfish *Faxonius immunis* in several German populations in a few years; Herrmann et al., 2022). Moreover, Monde et al. (2017) have documented efficient consumption of *M. tuberculata* by *C. quadricarinatus* under experimental conditions. However, *M. tuberculata* often co‐occurs with *T. granifera*, as well as native snails, in Martinique rivers, and evaluating how predation affects both species would be interesting.

Among those traits that may mediate the predator–prey interaction is the body size of both predator and prey. Both crayfish and snails grow indefinitely, and certain size classes of snails may be inaccessible to or less preferred by small crayfish, serving as “size refuge” (Ebbestad & Stott, 2008). Crayfish sex may also play a role as male crayfish are generally larger than females due to a larger number of molts as adult (Grandjean et al., 1997; Saoud et al., 2013; Stein, 1976), potentially leading to a greater efficiency in predation. Another important trait that may mediate the interaction is the ability of snails to mount behavioral responses to predator presence (or cue of), such as crawling out of water or burying into the sediment (Mathers et al., 2022; Ueshima & Yusa, 2015), but these aspects requires more work. Finally, the shell characteristics may also have an importance, as a snail with a rounder shell, therefore harder to handle for a crayfish than a thin, elongated shell, could more easily escape predation (Krist, 2002).

We studied the predation behavior of the crayfish *C. quadricarinatus* on three snail species occurring in Martinique (the two introduced Thiarids *T. granifera* and *M. tuberculata*, and the local species *N. punctulata*). We conducted several experiments to address the following questions: (i) Are the snail species equally exposed to predation by crayfish, depending on their shell morphology? (ii) Does predation efficiency increase with crayfish size and sex? (iii) Are some snail size classes relatively protected from predation (size refuge)? (iv) Do snails exhibit antipredation behavior (crawling out or burying) in the presence of predator cues? Based on the literature, our field observations, and species morphology, we expected a stronger predation pressure on snails with elongated shells (Krist, 2002), increased in the presence of large‐sized crayfish (and possibly by male, often larger than females). We also hypothesized not only that larger shells protect snails from predation (referred to as “passive escape,” since behavior is not directly involved), but also that two active behaviors can also play this role (“active escape”): burial in the sediment in *M. tuberculata* and crawling‐out in *T. granifera*. We finally discuss our results in the context of the management of freshwater IAS in Martinique. Our study provides new insights into predator–prey relationships, focusing on a predatory crayfish whose range is expanding rapidly in tropical fresh waters, but whose effects on its prey (here snails) remain poorly documented. Importantly, we analyzed the impact on several prey species, which is rarely done in experimental studies, along the whole predation sequence, from encounter to prey survival. We show that crayfish size and sex play a significant role, but that snail survival also depends on species and shell morphology. Snail species can also different active behavior to avoid predation.

## 2 MATERIALS AND METHODS

### 2.1 The species studied

**FIGURE 1:**
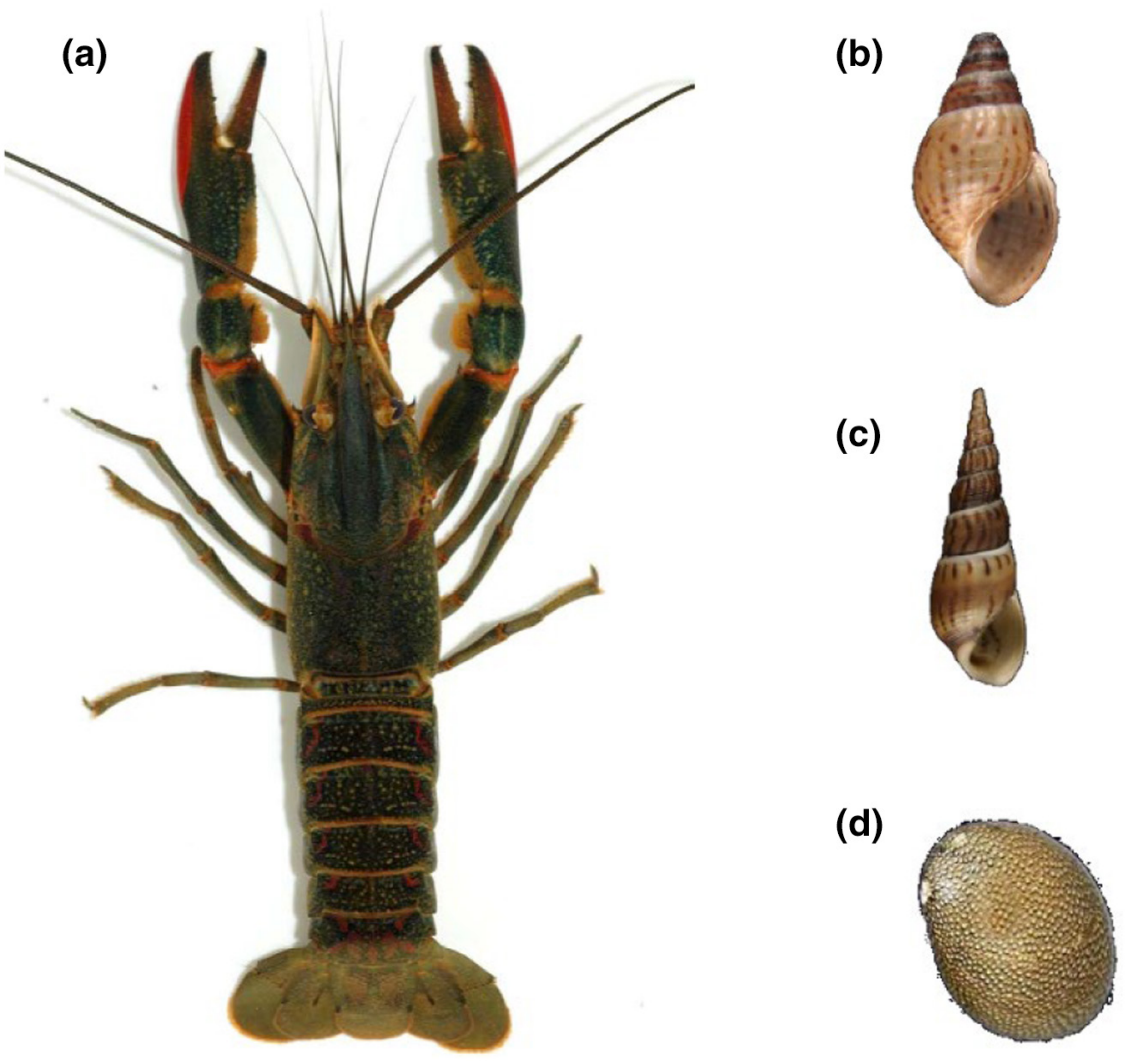
Pictures of the four species studied. (a) The invasive crayfish *Cherax quadricarinatus*; (b, c) the introduced snails *Tarebia granifera* and *Melanoides tuberculata*; (d) the native snail *Neritina punctulata*. Snail pictures from Delannoye et al. (2015).

Martinique is a French volcanic island (1128 km^2^) harboring a dense hydrographic network, with 70 permanent rivers, fed by numerous small tributaries. Hydrology is dominated by the rainfall regime, with a marked rainy season (*ca.* September to December). These rivers host a macrobenthic fauna of crustaceans, mollusks, and fish, of which at least 20 species (on 40–50) have been introduced over the last half‐century (Lim et al., 2002; Pointier, 2008). We considered here three mollusks species, *T. granifera*, *M. tuberculata*, and *N. punctulata* (Figure 1). The first two species belong to the Thiaridae family and were introduced in Martinique in the late 1970s and early 1990s, respectively (Delannoye et al., 2015; Facon et al., 2008; Pointier et al., 1998). They represent by far the dominant component of mollusks in Martinique freshwaters, both occurring in the majority of watersheds – their densities vary in time, but can reach 10,000 ind./m^2^ in both species (Facon et al., 2008; Samadi et al., 1997; P. Jarne & P. David, unpublished observations). *Neritina punctulata*, belonging to the Neritidae family, is naturally present in Martinique and frequently found in the lower reaches of rivers (Delannoye et al., 2015; Pointier, 2008). Local Gobiidae (small‐sized fish) may be occasional predators on these snails (Nordlie, 1981), but the only serious predator is the single crayfish from Martinique, *C. quadricarinatus* (Figure 1). Originating from Australia, it was introduced in the early 2000s for aquaculture purposes and then colonized Martinique rivers around 2010, through escape or intentional releases and now occupies more than 20 rivers, including the six main watersheds, in some cases at high densities (with catch per unit effort >10; Baudry et al., 2020, 2021). It is unlikely that the snail species studied have been confronted to *C. quadricarinatus*, given that the later originates from Northern Australia (Haubrock et al., 2021), while the introduced snails are of African/Southern Asian origin (Delannoye et al., 2015). Moreover, they have been introduced in Martinique several decades prior to the crayfish invasion. This overall suggests that snails were “naive” with regard to crayfish predation prior to the rise of *C. quadricarinatus*. All these species are easy to maintain in the laboratory under standard conditions.

### 2.2 Species sampling and maintenance

The crayfish were captured during two field sessions at the beginning of the study in January 2022 and just before the *Prey behavioral responses* experiments (see Figure S1 for a timeline of the whole study). Crayfish sampling was performed in three sites (Galion river at Bassignac, 14°43′54″ N/60°59′4″ W; Lézarde river at Saint‐Maurice, 14°40′11″ N/61°0′1″ W; Coulisses river at Saint‐Esprit, 14°33′29″ N/60°56′24″ W; Figure S2), previously surveyed by Baudry et al. (2021). Individuals were captured using traps (80 × 25 × 25 cm, 1 × 1 cm mesh), baited with cat meatballs. Traps were placed in turbid and deep pits, the preferential habitat of *C. quadricarinatus*, for 24 to 72 h (see Baudry et al., 2020). The captured individuals (70 females and 65 males) were then brought back to the laboratory at the IFREMER station (Le Robert, Martinique). Prior to and between experiments, they were maintained in individual pots (3 L) stabilized with floating board set in 500 L tanks (Figure S3) and fed with small carrot pieces. This device allows each individual to have its own space, avoiding direct interactions between individuals, including fights, mutilation, and cannibalism, but sharing water at tank scale. Crayfish were placed in four identical 500 L tanks, with males and females haphazardly distributed. Pots and tanks were numbered, ensuring that crayfish were individually identified. Randomization was ensured by regularly moving individuals among tanks, thus avoiding possible habituation to a given tank and set of crayfish. The sampling site was not taken into account in the experiments.

Snails were collected during the same two field sessions than crayfish, for a total of more than 400 individuals of each species. Sampling of *M. tuberculata* and *T. granifera* was carried out manually or with a dip net in four sites (the already‐mentioned Galion river at Bassignac and Coulisses river at Saint‐Esprit; Petite Pilote river at Pont Madeleine, 14°29′46″ N/60°54′18″ W; Grande Pilote river at Lowinsky, 14°31′0″ N/60°52′55″ W; Figure S2). These sites harbor both the crayfish (Baudry et al., 2021) and the targeted snail species (unpublished data from P. Jarne & P. David). *Neritina punctulata* was sampled using the same method in two sites, Carbet river at Aqwaland (14°42′54″ N; 61°10′7″ W) and Lorrain river at Seguineau (14°49′31″ N; 61°2′35″ W), and we end up with *ca.* 200 individuals. All snails were brought back to the IFREMER laboratory and were maintained (each species separately, all sites combined) in 60 L aquariums, equipped with lids to avoid snail escape. Snails were fed with leaf litter collected in the field.

All aquariums were filled with conditioned tap water and experimental conditions were 25–27°C, pH = 7.5, and 12/12 h photoperiod, close to natural conditions. Water was oxygenated continuously with a centralized bubbler (Figure S3) and changed every week after thorough cleaning (brushing and rinsing with tap water and drying for several days). For the experiments, we used a dedicated set of aquariums. Aquariums were cleaned (see before) after each experiment, and each experiment was initiated with clean water. Note that Thiarid snails readily reproduce under such laboratory conditions, providing G1 individuals that were used in those experiment requiring very small individuals (<*ca.* 4 mm). Such individuals are indeed not easily captured under natural conditions.

### 2.3 Pre‐experimental treatment and measurements

Several measurements (to the nearest mm), classically used in crayfish studies (Akmal et al., 2021; Grandjean et al., 1997), were taken, from each captured crayfish to the nearest mm using a digital caliper: the total length (TL), the carapace length (CL), the main claw length (MC), the main claw width (CW) at finger base, and the abdomen width (Abd; Figure S4). Crayfish were individually monitored, and each individual was used less than three times in the experiments described next, and never experiencing the same conditions to avoid habituation to experimental tests (Haddaway et al., 2012). Before each experiment, individuals were again sexed (males exhibit a red spot on claws and copulatory appendices), measured, and weighed (to the nearest g). Individuals were starved for 4 to 5 days prior to each experimental test.

Four classical measurements were taken on snails captured during the first sampling period (250 individuals of each Thiarid species and 200 *N. punctulata*), using a digital caliper (see Chiu et al., 2002; Samadi et al., 2000): the shell length (SL) and width (SW), as well as the length (AL) and width (AW) of the aperture (Figure S4). These measurements were used for the morphological analysis (see the Section 2.5. Statistical analysis section), and we then considered only the shell length before each experiment. *Melanoides tuberculata* and *T. granifera* are parthenogenetic species, essentially constituted of female individuals, such that sex is not an issue in our experiments. *Neritina punctulata* considered here do not exhibit sexual dimorphism, and sex was not considered. Each experimental test was preceded by a 48‐h acclimation phase, during which snails were set together without predator or predator cue.

### 2.4 Predator–prey experiments

We conducted three sets of experiments to investigate various aspects of snail–crayfish interactions, including snail mortality under predation and snail reaction to predation cue.

#### 2.4.1 Predation efficiency

This experiment aimed at investigating the influence of crayfish sex and size on predation efficiency when several prey species are available. Thirty replicate aquariums (55 × 19 × 39 cm, half‐filled with conditioned tap water) were set up (Figure S3), each including 25 snails, i.e., 10 *T. granifera* (SL: 8–29 mm), 10 *M. tuberculata* (SL: 8–22 mm), and 5 *N. punctulata* (SL: 3–12 mm). Less individuals of the latter species were included because preliminary observations indicated limited predation. In nine aquariums, a female crayfish (TL: 51–137 mm) was introduced 48 h later. Eleven aquariums received a male crayfish (TL: 95–172 mm). Ten aquariums received no crayfish, serving as mortality control. Crayfish were removed after 48 h, and surviving mollusks were counted and measured.

#### 2.4.2 Minimum and maximum prey size

This experiment aimed at estimating the minimum and maximum size of snails (*T. granifera* and *M. tuberculata*) that can be predated by crayfish. Trials were conducted separately for two species and two size classes, with 10 replicates per trial (40 in total). Each replicate included five snails, for a total of 200 studied individuals. Trials were conducted in the same type of aquariums as those described earlier. Snails were introduced in aquariums for 48 h prior to testing, and trials last for 24 h. Tests on both minimum and maximum sizes included five snails. For the minimum size, we used individuals with SL < 5 mm. For maximum size, the size range (SL: 19–30 mm in *T. granifera* and 15–21 mm in *M. tuberculata*) was based on observations from natural populations. A single crayfish was introduced per aquarium – we did not evaluate the effect of crayfish sex in this experiment, and individuals were drawn at random in a group with a balanced sex ratio (TL: 77–138 mm). The crayfish were removed after 24 h, and snail mortality was evaluated. We initially intended to then sequentially increase (for minimum size) and decrease (for maximum size) the size of snail prey, but all snails with length smaller than 5 mm were eaten in the first trials and only three (out of 100) larger than 15 mm (confronted with small crayfish, between 77 and 90 mm) survived, and we therefore stopped the experiment.

#### 2.4.3 Prey behavioral responses

The last set of experiments aimed at analyzing the predator avoidance behavior of Thiarids (*T. granifera* and *M. tuberculata*) in response to different stresses (predator cue, predator presence, and dead conspecifics). Each stress was tested separately in aquariums filled with 5 cm of sand gravel type substrate and 20 cm of water. No size class (for crayfish and mollusks) or sex restrictions (for crayfish) were applied, and the individuals used in this experiment reflect the size classes mostly found in Martinique rivers, i.e., adult snails with SL longer than 10 mm and crayfish between 65 and 150 mm. This means that we assumed, as no direct contact occurred, that crayfish individuals of all sizes would induce the same response in snails through the same signal. For the three tested stresses, the experimental snails were set haphazardly on the aquarium sandy bottom, and we monitored their position, either as “on the wall” (meaning vertical migration) or “buried in the sand.” This was done every 15 min for 1 h 30 min after their introduction. In the three sets of experiments, 20 snails were introduced per aquarium (*T. granifera*, SL: 10–25 mm; *M. tuberculata*, SL: 12–21 mm), and the test was performed in 10 aquariums per species. Ten aquariums per species served as control: the same protocol was applied without crayfish cue. Note that in the absence of stress, individuals of both species generally forage on the aquarium ground (sand). Each set of experiments therefore included 40 aquariums (120 in total).

Chemical stress – prior to the test, a single crayfish was placed for 7 days in each experimental aquarium, leaving ample time to emit chemical signals, and removed before introducing the 20 snails.

Physical presence of crayfish – A single crayfish was introduced per aquarium for 30 min and then caged, preventing any direct crayfish–snail contact (Figure S5), before introducing the 20 snails. Each crayfish was placed in a pot, allowing it to be mobile and possibly adding further cues of its presence to snails, such as water movement and sound.

Dead conspecifics – five conspecific individuals were manually crushed and set in each aquarium, before introducing the 20 snails. Snails are known to be sensitive to this stimulus, with responses highlighted in many studies in the literature (Covich et al., 1994; Hayashi & Sugiura, 2021).

### 2.5 Statistical analysis

All statistical and graphical analyses were performed in the R environment (R v4.0.2; R Development Core Team, 2023). Before each statistical treatment, when appropriate, data normality and variance homogeneity were verified using Shapiro–Wilk and Bartlett tests, respectively.

We first analyzed morphometric variation between sex (crayfish) and species (snails) and searched for correlations between morphometric variables based on a principal component analysis (PCA) using the *FactoMineR* (Lê et al., 2008) *and factoextra* (Kassambara & Mundt, 2020) packages. The eigenvalues were extracted using the *get_eigenvalue()* function and the correlation between the different morphometric variables was investigated using the *fviz_pca_var()* function. Individuals were projected on the factorial axes using the *fviz_pca_ind()* function. Sexual dimorphism in crayfish morphology was analyzed using a linear mixed model (LMM), regressing main claw size (MC) on total crayfish length (TL) (as random effect) considering sex as a fixed effect. Variances in TL measurements from female and male crayfish were compared using a Bartlett's *K* ^2^ test. MC/TL ratio was calculated in crayfish and sex influence was tested using a Student *t* test with unequal variance distribution. Interspecific variation in snail shell shape was analyzed using the classical SW/SL ratio (±SD [standard deviation]) which evaluates shell “rotundity.” Spherical shells have ratios tending toward 1, while low ratios indicate elongated shells. The influence of SL on SW was analyzed based on a LM and an analysis of variance (ANOVA), considering species (*T. granifera*, *M. tuberculata*, or *N. punctulata*) as fixed effect.

Survival in the first experiment (“Predation efficiency”) was analyzed using a generalized linear mixed model (GLMM) with a binomial distribution (number of dead vs. alive snails) and logit link, considering three fixed effects (crayfish sex, crayfish size, and snail species), adding aquarium as a random effect to account for overdispersion. Significance tests were made using likelihood ratio tests (LRTs) and results were expressed with a chi‐squared test (*χ* ^2^) and *p* value. The behavioral response of snails to the three stresses in these experiments (“Prey behavioral response”), by comparison to the controls (without predator), were also analyzed using a binomial GLMM **–** we indeed observed two behaviors in each species per experimental condition: crawling on the ground and on the wall in both controls and in *T. granifera* with predation, and burying in the sand and crawling on the wall in *M. tuberculata* with predation. Snail species was considered as a fixed effect, and we added aquarium as a random effect as previously. Preliminary data analysis indicated that snail response (e.g., ratio of individuals on the wall) reached a plateau after 1 h (out of 90 min observation), and the GLMM was run on the values at this time point. Results were reported with the associated chi‐squared tests for all stresses versus control condition comparison, and then each stress (chemical, physical, and dead conspecifics) was compared to control condition, using a *z* test (*z*).

## 3 RESULTS

### 3.1 Morphometry

**FIGURE 2:**
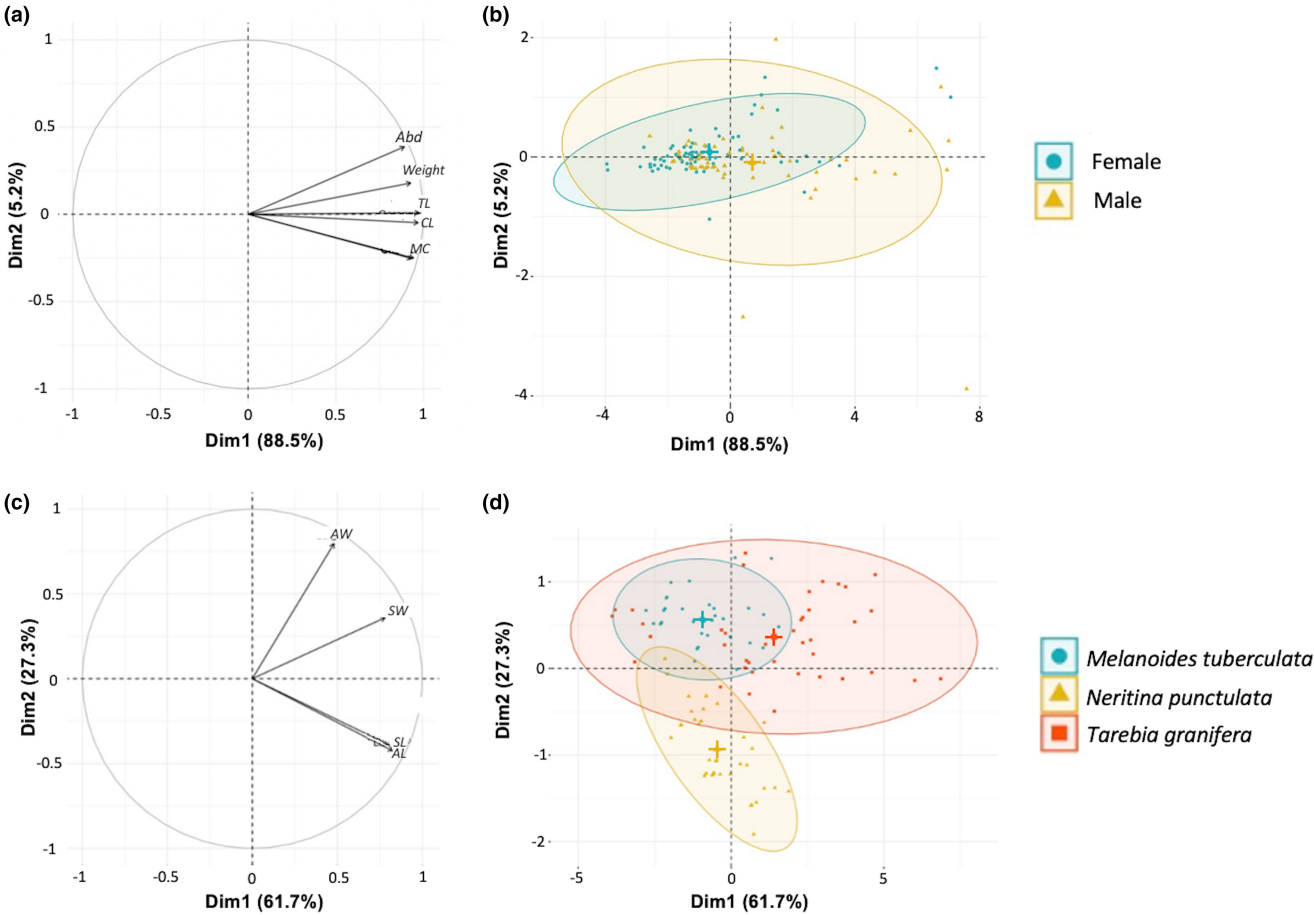
First two axes of a principal component analysis on morphometric measurements in crayfish and snails. (a) First factorial plane in crayfish with 88.5% of variation on axis 1. (b) Distribution of crayfish individuals according to sex (female as blue dots and male as orange triangles) with concentration ellipses (0.95 confidence interval). (c) First factorial plane in snails with 61.7% of variation on axis 1. (d) Distribution of snail individuals according to species (*Melanoides tuberculata* in green, *Neritina punctulata* in yellow, and *Tarebia granifera* in red) with concentration ellipses (0.95 confidence interval). Centroids for each concentration ellipses were highlighted with a cross, of the dedicated color.

The morphometric analysis in crayfish indicated a strong sex dimorphism for all six measurements (Table S2A), especially regarding the main claw size (Figure S6, Table S1). For example, the mean size in males was 123.6 mm versus 94.5 mm in females, with males 2.5 times heavier than females. The PCA showed a positive correlation between all measurements (Figure 2a), with notably almost 90% of the variance expressed on axis 1. Unsurprisingly, TL shows the strongest correlation with this axis.

A sex differentiation in morphometry is also clearly visible (Figure 2b), with males showing more variation than females and reaching larger maximal sizes (Bartlett's *K* ^2^ = 17.085, *p* < .001). Interestingly the correlation between MC and TL (*p* < .001 in both sexes; Figure S6, Table S1) did not exhibit the same slope and is steeper in males than in females, with ratio MC/TL significantly lower for females than males (respectively 0.29 vs. 0.35; *t* = −5.9, *p* < .001).

In snails, almost 62% of the explained variance was captured by the first principal component, and 27% by the second (Figure 2c). PC1 was positively correlated to all measurements (reflecting variation in overall size), while PC2 was a shape component opposing relatively wider shells and apertures to relatively elongated ones (positive correlation with SW and AW; negative with SL and AL). On the first factorial plane, Thiarids were clearly separated from *N. punctulata* (elongated vs. rotund shells), as were *T. granifera* and *M. tuberculata*, though to a lesser extent (Figure 2d). SL was significantly correlated to SW (*F*_1,499_ = 1478.18, *p* < .001), with strong species differences (*F*_1,499_ = 640.67, *p* < .001): *M. tuberculata* indeed displayed a low shape index (0.31 ± 0.05), with a homogeneous distribution of points, reflecting an elongated and narrow shape, while *N. punctulata* presented higher values (0.75 ± 0.07), reflecting a rather spherical shape (Figure S7). *Tarebia granifera* had an intermediate shape (0.46 ± 0.08), reflecting a moderately elongated and stocky shape (Figure S7).

### 3.2 Crayfish predation and snail survival

**FIGURE 3:**
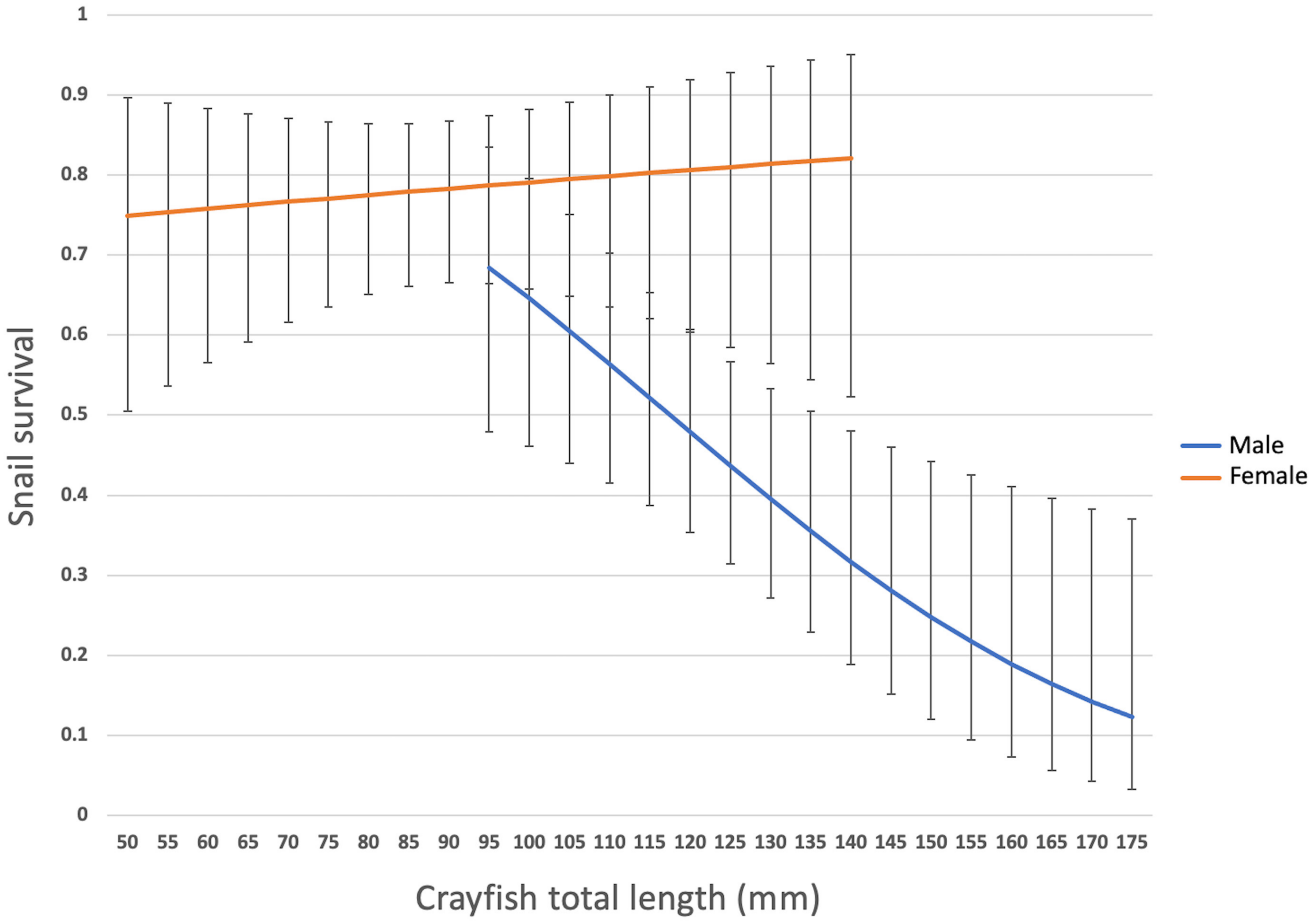
Survival of Thiarid snails (*Melanoides tuberculata* and *Tarebia granifera*) in the presence of the crayfish *Cherax quadricarinatus* as a function on crayfish size (TL) and sex (male in blue and female in orange), predicted by the GLMM. Standard error bars were calculated from the model in Table 1. The range of sizes represented for males and females is the one observed in our samples.

No snail died in the control treatment. The mean snail survival proportion, in the presence of crayfish, was 0.64 ± 0.21 (SD), but varied with crayfish sex and size and with snail species. With female crayfish, survival proportion was 0.78 ± 0.10 over all snail species, much higher than the value observed with male crayfish (0.53 ± 0.22). It was also significantly higher in *N. punctulata* (0.815 ± 0.215) based on a LRT (*p =* .004) and the GLMM on survival was therefore run only in Thiarids (Table 1). No effect of the Thiarid species alone, neither combined with crayfish size, crayfish sex, or with both crayfish size and crayfish sex was found (Table 1). Nevertheless, we detected an interaction between crayfish size and sex (Table 1, Figure 3), and therefore analyzed data separately according to crayfish sex. No variation in snail survival occurred with crayfish size in females, while survival was on average lower and markedly decreased with size in the presence of males (Table 1), with an estimated survival decreasing from *ca.* 0.7 to almost 0.1 over the size range of males (Figure 3).

**TABLE 1:** Results of the generalized linear mixed model (GLMM) performed to investigate the influence of three fixed effects (crayfish sex, crayfish size, and snail species), adding aquarium as a random effect, on snail survival.

Data	Fixed effects	$\chi_{3 df}^{2}$	*p*
All	Crayfish size × crayfish sex × prey species	0.98	.32
	Crayfish size × crayfish sex	4.32	.037
	Crayfish size × prey species	1.13	.29
	Crayfish sex × prey species	0.1	.75
	Prey species	0.77	.38
Crayfish (male)	Crayfish size	5.68	.017
Crayfish (female)	Crayfish size	0.16	.68

*Note*: Data include only Thiarids as *Neritina punctulata* was not efficiently predated. Likelihood ratio test (LRT) is provided for Thiarids survival (both *Melanoides tuberculata* and *Tarebia granifera* combined). Given the significant interaction between crayfish size and sex, and the lack of prey species effect, we tested the crayfish size effect separately for each sex, combining the two prey species.

We then tested whether snails can passively escape predation when they are too small or too large (“size refuge”). In the absence of shelters, no such escape seems possible: in both trials, all snail individuals were killed (“minimum size” tests) and only three stay alive (“maximum size” tests) within the 24 h of the trial. These three snails (>15 mm) had indeed been confronted to relatively small crayfish (<90 mm) that tried to attack their shell, but failed to consume them. Considering this attack behavior and that all crayfish over 110 mm attacked and killed snails (>15 mm), we tentatively conclude that no “absolute” refuge size exists. Qualitatively, we observed that crayfish predation strategy seemed size dependent (for both prey and predator). Mollusks smaller than 5 mm were all fully crushed and consumed by crayfish of all size. Additionally, crayfish over 110 mm were able to crush the shell and consume large snail prey (>15 mm) by reaching directly into the snail body, whereas smaller crayfish only left traces of attack on shells, with sometimes the shell emptied, after the operculum was torn off. When we were able to observe attacks, smaller crayfish tended to grasp the shell and use their mandibles to pull out the soft parts (“sucking” the body).

### 3.3 Prey behavioral responses

**FIGURE 4:**
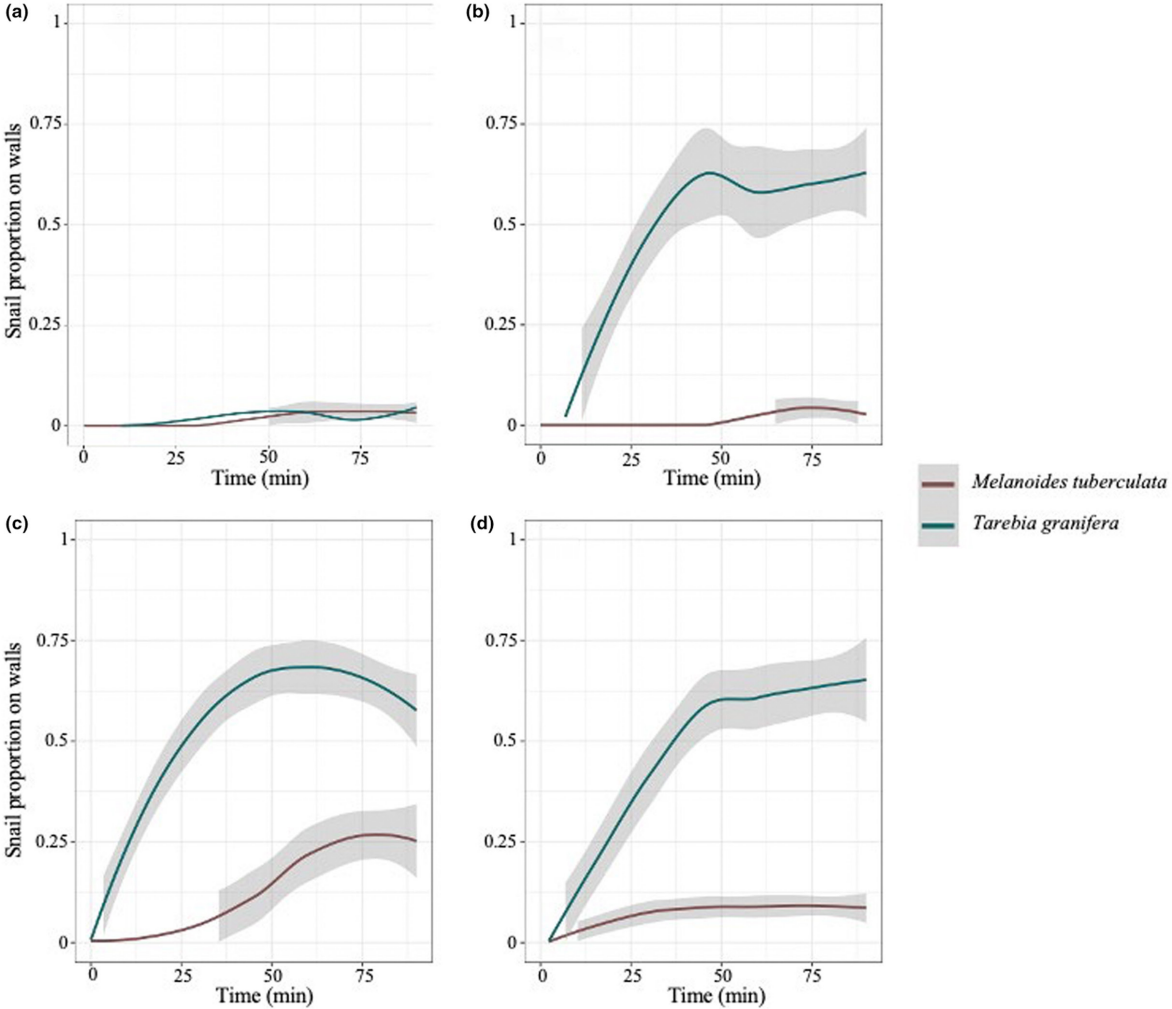
Proportion of Thiarids snails (*Melanoides tuberculata* in red and *Tarebia granifera* in green) on the aquarium walls as a function of time in the “prey escape behavior” experiments. (a) Control condition (no stress). (b) Crayfish odor in the aquariums as stress. (c) Caged crayfish as stress. (d) Presence of dead (freshly crushed) conspecifics as predator cue. Regression curves were smoothed using the LOESS method (0.95 CI in gray shade).

The strategies adopted by snails in these trials are presented in Figure 4. In the control condition (Figure 4a), snails from both species mostly forage on the surface of the substrate and rarely crawled on the wall (e.g., one individual in *T. granifera* at 30 min, two in both species at 60 min, two in *M. tuberculata* at 75 min, and one in both species at 90 min). In the presence of predation cues or caged predator, *T. granifera* individuals mostly climbed up the walls, though a few keep crawling on the ground. The answer in *M. tuberculata* was mostly burrowing in the sand, with some individuals climbing on the walls. “Snails on the wall” was therefore used to quantify snail response in both species (Figure 4). In the three stress conditions, *T. granifera* tended to escape the aquarium ground, while *M. tuberculata* buried into the sandy ground (Figure 4b–d, Table 2). The behaviors reached a plateau after 60 min (Figure 4), and considering species differential behavior we analyzed species separately using values at this time point. We observed a marked effect of stress for both species, but the two species behaves differentially under the three stresses (Figure 4, Table 2): *T. granifera* seemed to react significantly to all stresses compared to control condition, while *M. tuberculata* significantly climbed up along the wall only in the presence of the crayfish (physical stress) with no effect for chemical and dead conspecifics stress (Table 2).

**TABLE 2:** Results of the GLMM investigating the effects of species (*Tarebia granifera* or *Melanoides tuberculata*) and stress (chemical cue, physical presence of crayfish, and dead conspecifics) on the snail behavioral responses, after 60 min of experiment.

Data	Fixed effects	Statistics	*p*
All	Species × stress	$\chi_{3 df}^{2}$ = 22.45	<.001
*Tarebia granifera*	Stress	$\chi_{3 df}^{2}$ = 57.07	<.001
	Chemical vs. control	*z* = 7.198	<.001
	Physical vs. control	*z* = 8.512	<.001
	Dead conspecifics vs. control	*z* = 7.197	<.001
*Melanoides tuberculata*	Stress	$\chi_{3 df}^{2}$ = 21.69	<.001
	Chemical vs. control	*z* = 0.539	.59
	Physical vs. control	*z* = 4.009	<.001
	Dead conspecifics vs. control	*z* = 1.461	.14

*Note*: Because of the significant interaction, the effect of stress was tested separately in the two snail species. For each species, the different stress stimuli are compared to control condition using *z* test.

For the chemical stress, 16% of *T. granifera* individuals have migrated vertically along the wall after 15 min, and more than 50% after 45 min (Figure 4b). Conversely, most *M. tuberculata* individuals buried themselves very fast in the substrate until the end of the experiment (Figure 4b). For the stress induced by the physical presence of the predator (caged *C. quadricarinatus*), the movements observed in *T. granifera* are the same as in the previous experiment (Figure 4c). In this situation, some vertical movements were observed in *M. tuberculata* individuals, reaching a fraction of 25% of individuals after 90 min (Figure 4c). The responses under the last stress (dead conspecifics) were similar than with the other stresses in *T. granifera*, and intermediate between the responses to the two other stresses in *M. tuberculata*, with *ca.* 10% migrating up the walls, while the other stayed buried (Figure 4d).

## 4 DISCUSSION

We investigated the predator–prey relationship between the invasive redclaw crayfish (*C. quadricarinatus*) and several snail species co‐occurring in Martinique rivers (two invasive, *T. granifera* and *M. tuberculata*, and the endemic *N. punctulata*) through laboratory experiments. In the following section, we discuss our results following the temporal sequence going from crayfish–snail encounter to snail survival following encounter. More specifically, we first discuss escape strategies in snails that aim at avoiding confrontation with crayfish. We then consider how both prey and predator phenotypes (e.g., size) affect the outcome of the interaction, i.e., passive avoidance of predators by prey and efficient strategies in predators to detect and consume prey. Third, we discuss differential snail survival depending on both snail species and crayfish size and sex. These elements provide hints to explain/predict the collapse of snail populations in Martinique and the success of *C. quadricarinatus*.

### 4.1 Snail behavioral responses

Predation is one of the strongest selective pressures, and prey face this threat by developing antipredatory responses, first to reduce the probability of encountering predators (Alexander & Covich, 1991; Lakowitz et al., 2008). Active escape in slow movers such as gastropods is not an efficient strategy against mobile predators such as crayfish at a short distance, but snails can also detect cues (kairomones) at a long distance in the water. These signals can have different sources and nature: directly through the presence, current or past, of the predator, called “chemical signals,” or alarm signals from conspecifics killed by predation (Alexander & Covich, 1991; Lakowitz et al., 2008; Sih et al., 2010). Little is known about the detection mechanism of these cues, but a recent study highlighted that predator (the crayfish *Faxonius virilis*) recognition was rapidly learned by predator‐naive snails (*Lymnaea stagnalis*), but not transmitted to successive generations. Moreover, the time before responses was lower for predator‐experienced snails (Batabyal & Lukowiak, 2022; Tariel‐Adam et al., 2023).

Two snail behaviors have been described in experimental studies as a reaction to these signals: crawling out along the wall up to (or over) the waterline and burrowing into the sediment (Covich et al., 1994; Hayashi & Sugiura, 2021; Mathers et al., 2022; Ueshima & Yusa, 2015), with differences depending on both prey species and signal. For example, Ueshima and Yusa (2015) and Hayashi and Sugiura (2021) showed a quick response to an alarm signal from dead conspecifics, which was also promoted in the presence of the predator. These two distinct behaviors are also possibly linked to prey morphological characteristics: gastropod species that have a good ability to cling to a substrate (e.g., more spherical shells and powerful foot) tend to climb up this substrate, while species with elongated shells tend to bury into the sediment (Alexander & Covich, 1991; Turner, 1996).

Our results are consistent with these expectations. Snails from both Thiarid species were indeed able to detect the three signals (chemical from physical presence of crayfish, past presence of crayfish, and dead conspecifics), displaying behaviors differing from the control condition (without predator). At the beginning of the experiments, as well as during control experiments, individuals foraged on the sandy bottom. Any of the three stresses induced behavioral escape responses, as soon as 15 min after experiment initiation. The snail species with the more rotund and thick shell (*T. granifera*) immediately tried to climb up the aquarium walls, up to the waterline and sometimes above, while those with the more elongated shell (*M. tuberculata*) buried into the sand, in line with the abovementioned trend (Alexander & Covich, 1991; Turner, 1996). No significant difference was observed between the three stress conditions, except for the physical presence of *C. quadricarinatus*, that seemed to modify slightly the escape strategy of *M. tuberculata*, with more individuals (of both species) attempting to climb up the walls. Interestingly, these findings are consistent with field observations in Martinique rivers, where *T. granifera* are mostly found on rocks, often near the water surface and *M. tuberculata* more frequently in the sediment, or in dense algal beds (author's unpublished observations). These escape behaviors are also observed in these two species when the water current rises to extreme levels during flood (P. Jarne & P. David, unpublished observations), which occurs on a periodic basis in the tropical waters of Martinique. They therefore belong to generalist escape behaviors in freshwater snails serving as response to a wide range of environmental (biotic and abiotic) perturbation (Grudemo & Bohlin, 2000; Haubois et al., 2005; Orvain & Sauriau, 2002).

### 4.2 Shell morphology and predator attack

In addition to passive avoidance behaviors, gastropods can also rely on their armor (shell) as a passive way to defend themselves against predators (Vermeij, 1987). Indeed, the shape, thickness/resistance, and ornamentation of shells affect predation efficiency and gastropod survival (Krist, 2002). For example, a more rotund and smoother shell may make it harder for crayfish predators to handle it (but easier to swallow it for predators such as fish), and thicker shells is an efficient defense against crushing predators (Dickey & McCarthy, 2007; Miranda et al., 2016). As an example, Alexander and Covich (1991) showed a tendency for *Procambarus simulans* to abandon prey too long to handle (*Physella virgata*), because of their smooth structure lacking structural relief, in favor of prey easier to apprehend (*Physella viruta*), with a thinner and elongated spiral shell. We indeed observe that crayfish predation was far less efficient in *N. punctulata*, characterized by a rotund, smooth, and heavy shell, than in Thiarids, with more elongated, rugged, and light shells. The two Thiarid species also differ in shell shape, with more elongated shells in *M. tuberculata* than in *T. granifera* (Samadi et al., 2000). Shells are also thicker in *T. granifera* than in *M. tuberculata*, with a resistance to crushing in adults that is almost three times higher, as shown by Miranda et al. (2016) based on an experimental approach with an apparatus measuring shell resistance. These authors suggested that this might ensure an advantage in the former species when confronted with a crab predator. Using a more direct approach (i.e., with a living predator) and after confirming that the two species differ in morphology, we showed that shell resistance does not affect differentially survival in the two snail species when confronted with *C. quadricarinatus*. This predator seems strong and large enough to “easily” perform deadly attacks on the Thiarids present in Martinique – confirming its growing reputation of dangerous invasive species for freshwater macrobenthic faunas worldwide (Haubrock et al., 2021).

We also note that crayfish do not only rely on shell crushing to feed on snails. Alternative approaches are indeed used when crayfish are not strong enough (here, young females and juveniles). Crayfish use chelipeds (i.e., main claws) to crush snail shells, but they can as well empty shells by tearing off snail operculum with their mandible, while holding shells with their first two pairs of pereiopods. The latter (“sucking”) behavior has been observed in at least two decapods species, the crayfish *Faxonius rusticus* feeding on the freshwater mussel *Dreissena polymorpha* (Perry et al., 1997) and the Japanese spiny lobster (*Panulirus japonicus*) on two marine gastropods (*Chlorostoma argyrostoma* and *Lunella coreensis*; Takahashi et al., 1995). This is what we repeatedly observed here, especially in small crayfish (<100 mm) confronted with large snails (>15 mm). This strategy may explain why crayfish do not seem to be affected by differences in shell resistance between our two Thiarid species, since they can always remove them from the substrate and eat them without crushing. This is not possible with the native *N. punctulata* though, because the latter is both very difficult to crush and to remove from the substrate.

### 4.3 Predation pressure of *Cherax quadricarinatus*

Our results suggested a strong negative impact of *C. quadricarinatus* on snail survival, with a mean survival of 64% (compared to no mortality in controls), in our experiments. These results are consistent with those from previous studies which have reported strong effect of invasive crayfish on gastropods, for example, Hofkin et al. (1992) and Souty‐Grosset et al. (2016) with the crayfish *Procambarus clarkii*. Closer to our study, Monde et al. (2017) showed that predation by adult *C. quadricarinatus* may lead to no survival at all in *M. tuberculata* (100 individuals consumed over 7 days in controlled conditions). This high predation pressure in aquarium experiments may be linked to an absence of “refuge” size, demonstrated in our experiments: very small (<5 mm) or large (>15 mm) snails were indeed efficiently eaten by crayfish. The “refuge size” concept is very difficult to generalize, because it depends not only on both prey and predator species (whether crayfish or other taxa), but also on experimental or environmental conditions (see Covich, 2010). It might even not apply here, since *C. quadricarinatus* is an efficient predator given its large size, even for “normal” individuals, and at least two possible predation behavior (crushing and sucking; see before).

However, numerous factors can affect snail survival, including crayfish sex and size and the diversity of prey. In our experiments, male *C. quadricarinatus* were significantly more efficient predators than females (50% vs. 80% snail survival, irrespective of Thiarid species). This is consistent with previous results (Barbaresi et al., 2004; Garvey & Stein, 1993), and might be due to a strong sexual dimorphism in crayfish and different physiological processes between sexes (i.e., reproduction and growth) leading to different dietary requirements (Stein, 1976). Male crayfish grow faster than females and reach larger sizes, resulting in more frequent molting episodes and therefore higher food requirements (Jones, 1990). This has been observed in *C. quadricarinatus* (Haubrock et al., 2021) and in the cogeneric species *Cherax destructor* (Biro et al., 2014). The latter also display more active exploratory behavior leading to larger energy expenses. However, female redclaw crayfish may also have high food requirements during specific stages of the life cycle, especially reproduction (Ghanawi & Saoud, 2012; Saoud et al., 2012), but females were not at this stage in our experiments. A difficulty when assessing the sex effect on mortality is the strong sexual dimorphism in *C. quadricarinatus* – a classical issue in crayfish (see, e.g., Grandjean et al., 1997; Stein, 1976). Size indeed had a strong effect in males with snail survival falling from 70% in the presence of crayfish smaller than 100 mm to about 20% with large crayfish (>150 mm). No such effect was detected in females with snail survival approximately constant over the tested size range in crayfish (51–137 mm). A final factor that may affect predation efficiency here is the diversity of prey. Predator efficiency is often evaluated with a single prey species (Alexander & Covich, 1991; Nyström & Pérez, 1998), though prey choice is prevalent in nature. Not only dietary requirements may differ with ontogenetic stages, size, and sex in crayfish, but also aptitude to access to food and to consume it, explaining possible switches in diet along the life cycle (Guan & Wiles, 1998; Jackson et al., 2017) – *C. quadricarinatus* indeed is more of an omnivore than a strict carnivore. We detected clear differences in survival between *N. punctulata* and Thiarids, but not between the two Thiarid species (*T. granifera* and *M. tuberculata*), suggesting no prey preference. Whether this applies to other food sources should be further studied.

Overall, crayfish sex (and size in males) was the main predictor of Thiarid snail survival, probably because males have stronger claws and handle snails more efficiently. Further studies might analyze in more details the intake rate of crayfish as a function of food (snail) density – in other words, determine Holling's type functional responses (Holling, 1959) – according to sex, size, and prey diversity, as, for example, done by Haddaway et al. (2012) in two crayfish species (the native European species *Austropotamobius pallipes* and the invasive North American *Pacifastacus leniusculus*). This would be useful for better understanding not only the compromise between energy supply (assimilated food mass) and the energy cost induced for predation at individual level, but also the population dynamics of crayfish depending on available snail prey (Haddaway et al., 2012).

### 4.4 Caveats about our experiments

We conducted experiments to explore various sides of predator–prey interactions under artificial mesocosm conditions (e.g., small size mesocosms, no current, no rocks for hiding in snails). An important question, therefore, is how our results can be used to understand predator–prey interactions in Martinique rivers – a question that falls within the much broader framework of the respective interest of experimental approaches in the laboratory and under natural conditions in ecology (e.g., Diamond, 1983). We argue that our artificial conditions allow to evaluate the predation potential over a short period and its potential impact on dense snail populations when a new efficient predator is introduced. We considered a hiding option (burying in the sediment) and an escape option (climbing up the walls) for snails that have actually been observed in previous studies (Alexander & Covich, 1991; Turner, 1996) and in Martinique rivers (P. Jarne & P. David, unpublished observations). Including rocks, which can serve as refuges and are making a good part of the natural habitat, is an open possibility for further experiments. Note though that even when crayfish occur at high density, snails must forage to feed and risk being predated, so refuges are not absolute guarantees against predation. A second issue is that crayfish have access to other food sources in these habitats, presumably reducing predation risk by these omnivorous feeders. A last point is that the highest densities of both Thiarid species are observed near river bank, where the current is minimal. Testing predation in experimental conditions with current is therefore not necessarily compulsory. Further work is certainly required to assess the impact of crayfish on snail populations in Martinique, especially on the decline of *T. granifera* in many watersheds in the absence of other predators (P. Jarne & P. David, unpublished observations). Long‐term monitoring, modeling of population demography, and field experiments (e.g., using enclosures) are certainly required to progress in our understanding of these prey–predator relationships, as has been called in general for invasive species (see, e.g., Kueffer et al., 2013).

## 5 CONCLUSION AND IMPLICATIONS FOR CONSERVATION

Successive invasions have put together several IAS belonging to different trophic levels in the freshwater ecosystems in Martinique, the (predator) crayfish *C. quadricarinatus*, and the (prey) snails *M. tuberculata* and *T. granifera*. Both snail species showed escape capacity, which does not seem enough to avoid high mortality as soon as the snail is found by a hungry crayfish, especially in encounters with large male crayfish. Of course, our experimental setups probably resulted in higher predation rates than in nature, where the snails are not locked in a small aquarium together with their enemy. However, while Thiarids have been present for more than 40 years in Martinique, sometimes at very high densities, essentially limited by inter‐ and intraspecific competition (Facon et al., 2008; Pointier, 1993), our data definitely suggest that the arrival of *C. quadricarinatus* may change the game, possibly leading to demographic bust in these snails, and to demographic fast increase (boom) in this crayfish (see Strayer et al., 2017 for a review on boom–bust dynamics in invasive species). An open question is whether *C. quadricarinatus* may also impact local species, some of them having both low occurrence and low density (Delannoye et al., 2015; P. Jarne & P. David, unpublished data). Herrmann et al. (2022) indeed correlated the collapse of mollusks populations of several species, including endangered ones, in Germany, in the 7 years following the introduction of the invasive crayfish *F. immunis*. Considering these alarming observations and the relatively low richness of the Martinique freshwater ecosystems (i.e., not only 11 native mollusks species, but also 21 native fish and 13 native decapods species, some of which may serve as prey for *C. quadricarinatus*) (Delannoye et al., 2015; Lim et al., 2002), there is an urgent need to monitor the dynamics of redclaw crayfish. Invasive crayfish are well‐known to be difficult to control once the first populations have settled (Aquiloni et al., 2010; Gherardi et al., 2011). Strategies have to be developed to limit *C. quadricarinatus* expansion in Martinique and preserve the uninvaded streams, based on biomonitoring with early detection method (e.g., environmental DNA; Taberlet et al., 2018), to quickly act in case of new invasion (Baudry et al., 2021).

## AUTHOR CONTRIBUTIONS

**Thomas Baudry:** Conceptualization (lead); data curation (equal); formal analysis (equal); funding acquisition (equal); methodology (lead); software (equal); supervision (supporting); validation (equal); visualization (equal); writing – original draft (lead); writing – review and editing (equal). **Lola Millet:** Data curation (equal); formal analysis (equal); investigation (lead); validation (equal); visualization (equal); writing – review and editing (equal). **Philippe Jarne:** Formal analysis (equal); methodology (supporting); validation (equal); visualization (equal); writing – review and editing (equal). **Patrice David:** Formal analysis (equal); software (equal); validation (equal); visualization (equal); writing – review and editing (equal). **Frédéric Grandjean:** Conceptualization (supporting); funding acquisition (equal); methodology (supporting); project administration (lead); supervision (lead); validation (equal); visualization (equal); writing – review and editing (equal).

## CONFLICT OF INTEREST STATEMENT

The authors declare that they have no known competing financial interests or personal relationships that could have appeared to influence the work reported in this article.

## Supporting information


Data S1.

Data S2.

## Data Availability

All data generated or analyzed during this study are included in this published article (and its supplementary information files Data S1 and **S2**) and additional information and data are available from the corresponding author upon reasonable request.
