# Supplementary material for: Multiple invasions and predation: The impact of the crayfish Cherax quadricarinatus on invasive and native snails
Source: Ecol Evol. 2024 Apr 1;14(4):e11191. doi: 10.1002/ece3.11191 (PMC10985378; doi:10.1002/ece3.11191)
Supplement: Supplementary file 1 — Data S1. [file ECE3-14-e11191-s001.docx]

**Supporting Informations**

**Multiple invasions and predation: the impact of the crayfish *Cherax quadricarinatus* on two invasive Thiarid snails**

**Contents**

**Figures**

- **Figure S1.** Timeline of the whole study (January to May 2022).
- **Figure S2.** Sampling points prospected for crayfish and snails sampling.
- **Figure S3.** Setup and animal maintenance used in our experiments.
- **Figure S4.** Measurements performed in snails and crayfish.
- **Figure S5.** Experimental setup used for testing prey behavioral responses.
- **Figure S6**. Main claw length (MC) as a function of total length (TL) in *Cherax quadricarinatus*.
- **Figure S7.** Shape index values (SW/SL) in snail species.

**Tables**

- **Table S1.** Effect of total length (TL) of crayfish (*Cherax quadricarinatus*) on the main claw length (MC), considering sex (female and male) as a fixed effect.
- **Table S2**. Morphometric measurements in male and female *Cherax quadricarinatus* **(A)**, **and snails of the three studied species (B).**

**Figure S1.** Timeline of the whole study (January to May 2022). It began from laboratory preparation, which was followed by sampling, the first two sets of experiment, a second sampling period and the last set of experiments. Note the phase of thorough cleaning of experimental tools (e.g., aquariums) between each experiment and at the end of March. The number refer to section in the main text.

*
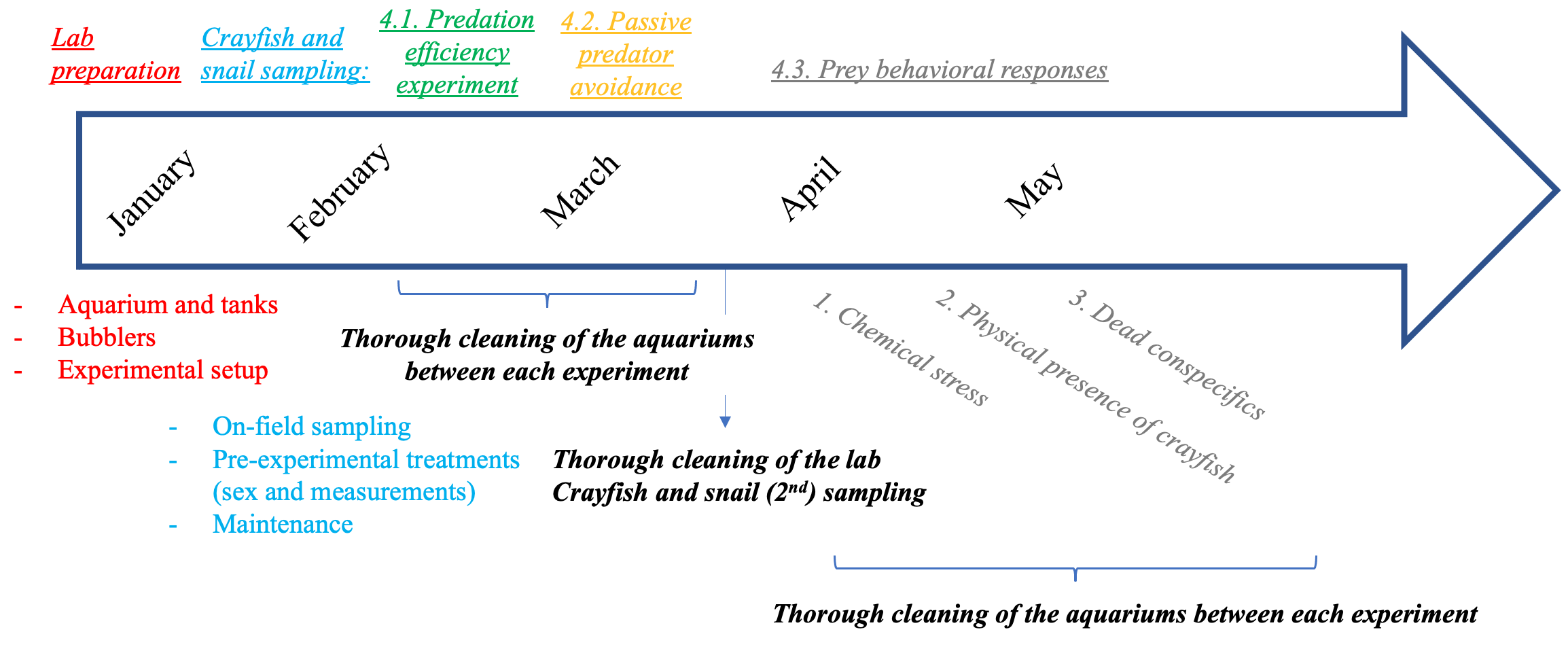
*

**Figure S2.** Sites that were sampled for crayfish and snail used in experiments. Three sites were chosen from Baudry et al. (2021) for crayfish, Bassignac in Galion river, Saint-Maurice in Lezarde river and Saint-Esprit in Coulisses river. Thiarids (both *Tarebia granifera* and *Melanoides tuberculata*) were captured in Bassignac in Galion river, Saint-Esprit in Coulisses river, Pont Madeleine in Petite Pilote river and Lowinsky in Grande Pilote river (where *Cherax quadricarinatus* was also found). *Neritina punctulata* were collected at Aqwaland in Carbet river and Seguineau in Lorrain river. More details on sites in main text.

**
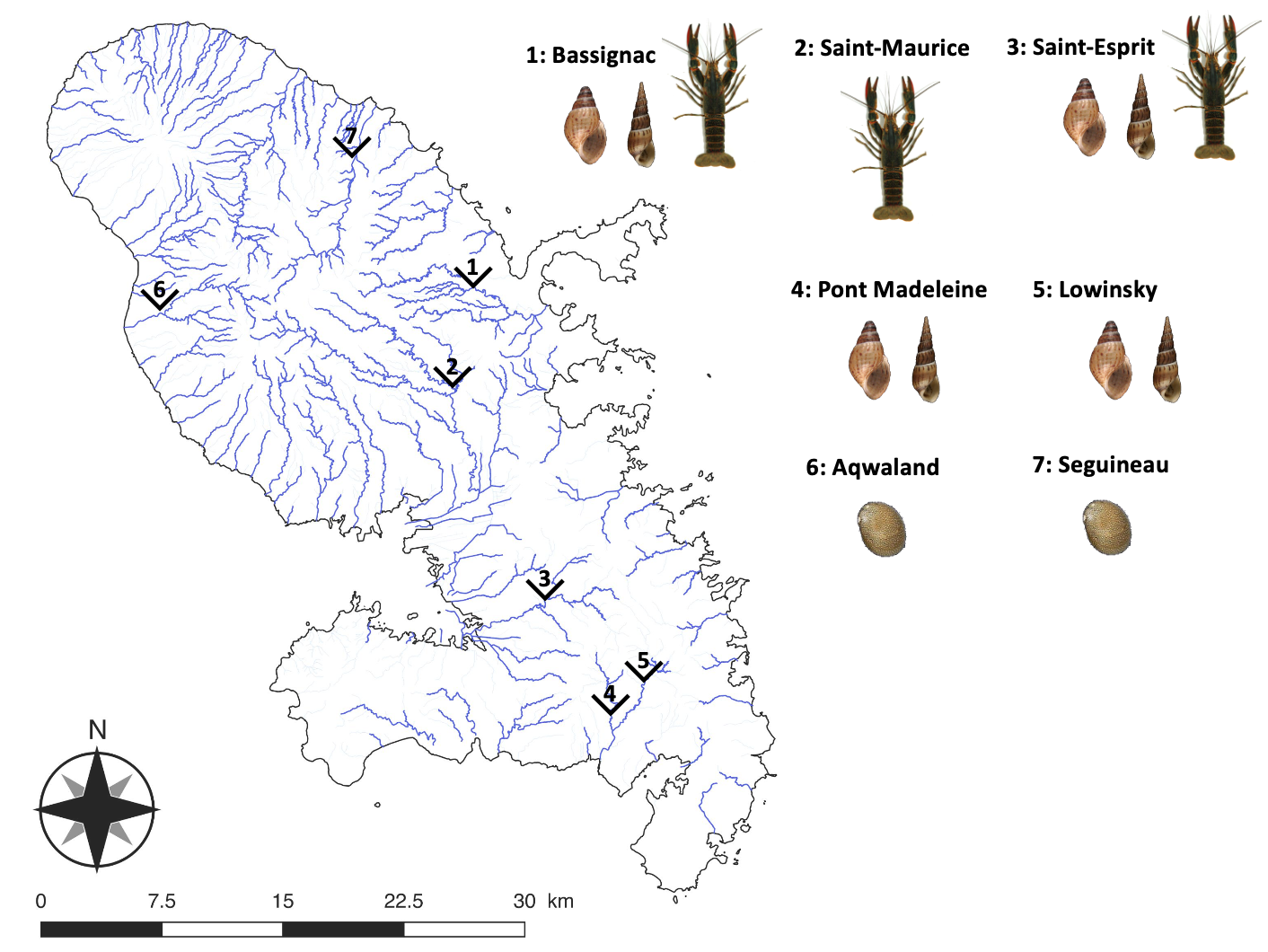
**

**Figure S3.** Setup and animal maintenance used in our experiments. Left: the aquariums in which snails were kept and experiments were conducted, set up on shelves. Aquarium disposition was randomized during experiment, and they were led under artificial light (12h / 12h photoperiod), without any external natural light. Right: board and pots, floating in a 500-L tank. Each pot hosts a crayfish. See text for more details.


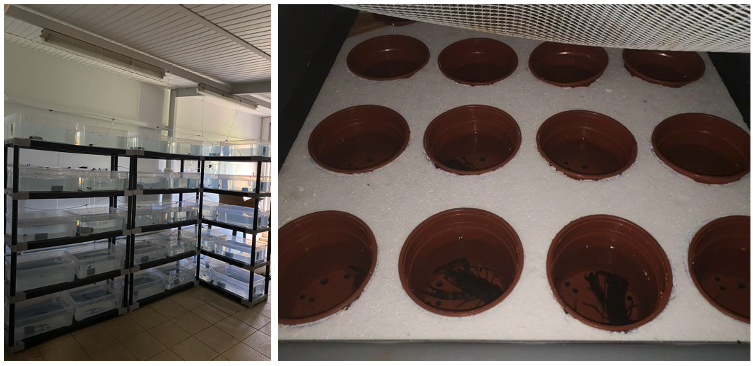


**Figure S4.** Measurements performed in snails and crayfish. Left: shell length (SL) and width (SW), aperture length (AL) and width (AW). The shell represented here belongs to *Melanoides tuberculata*. The shell of *Tarebia granifera* is very similar in shape, and therefore not represented. Right: total length (TL), carapace length (CL), main claw length (MC) and width (CW), and abdomen width (Abd) in the crayfish *Cherax quadricarinatus*.


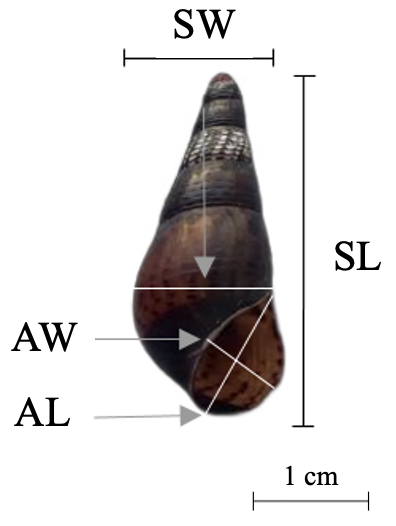

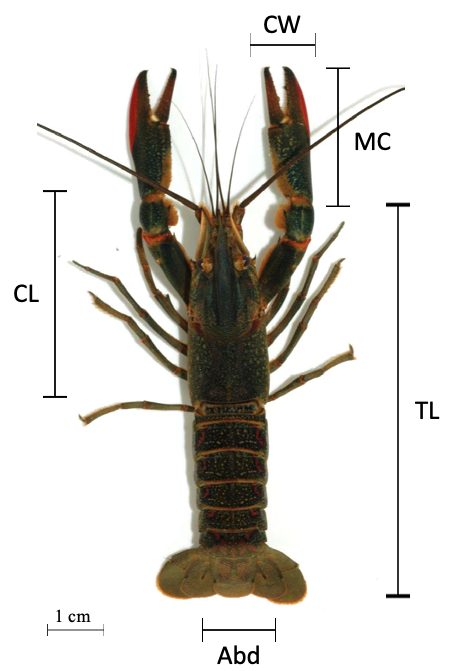


**Figure S5.** Experimental setup used for testing prey behavioral responses. See section 4.3 in main text. Crayfish were introduced in the aquarium 30 minutes before the beginning of the experiment, and then caged, using a gardening pot with holes. Holes were done to avoid any contact between crayfish and snails, but were sufficiently large to allow water flow.


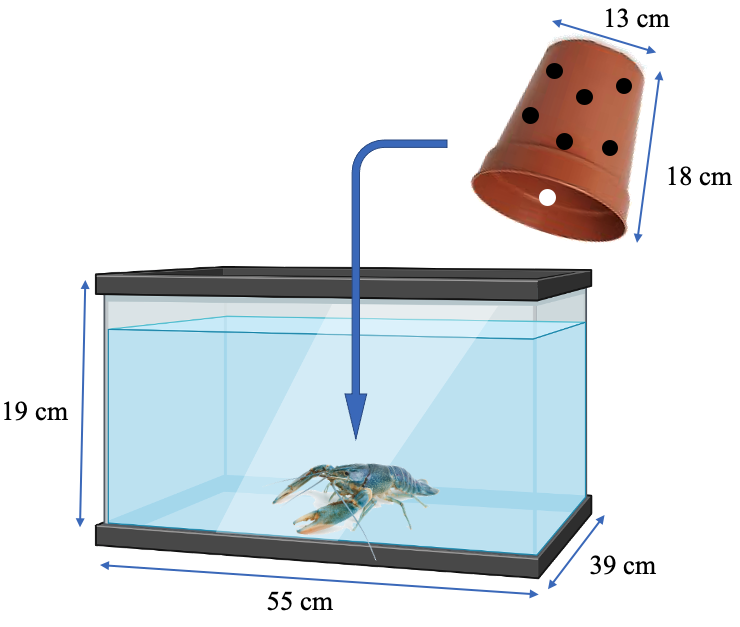


**Figure S6**. Main claw length (MC) as a function of total length (TL) in *Cherax quadricarinatus*. Each dot is an individual. Females are represented in red, and males in blue.


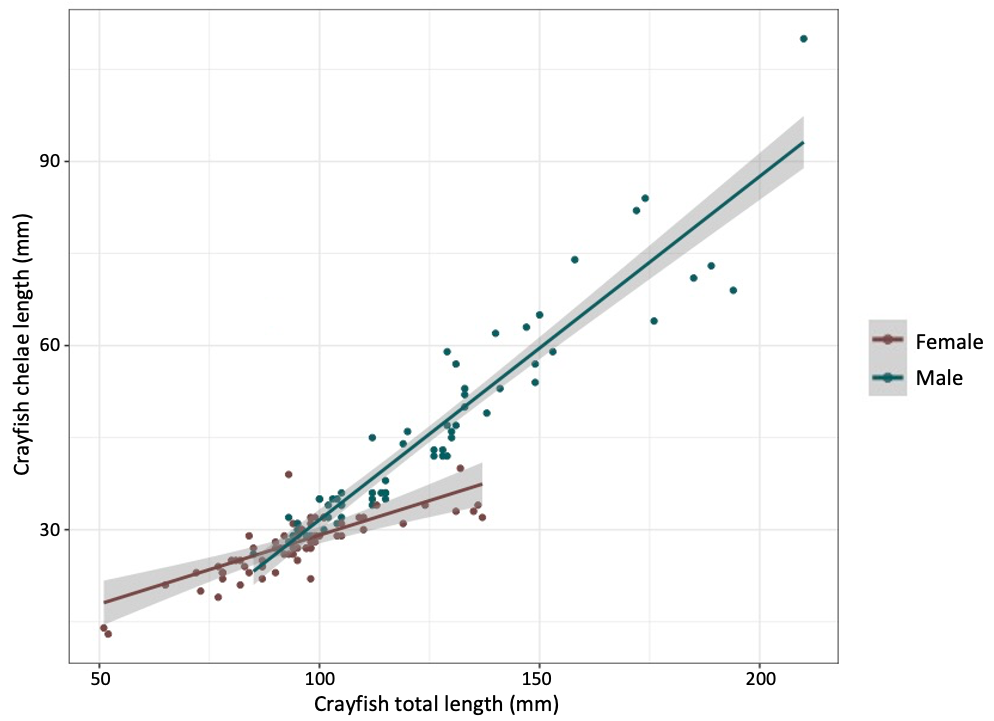


**Figure S7.** Shape index values (SW/SL) in snail species. *Melanoides tuberculata* in red, *Tarebia granifera* in blue and *Neritina punctulata* in grey. SL and SW are shell length and width respectively.


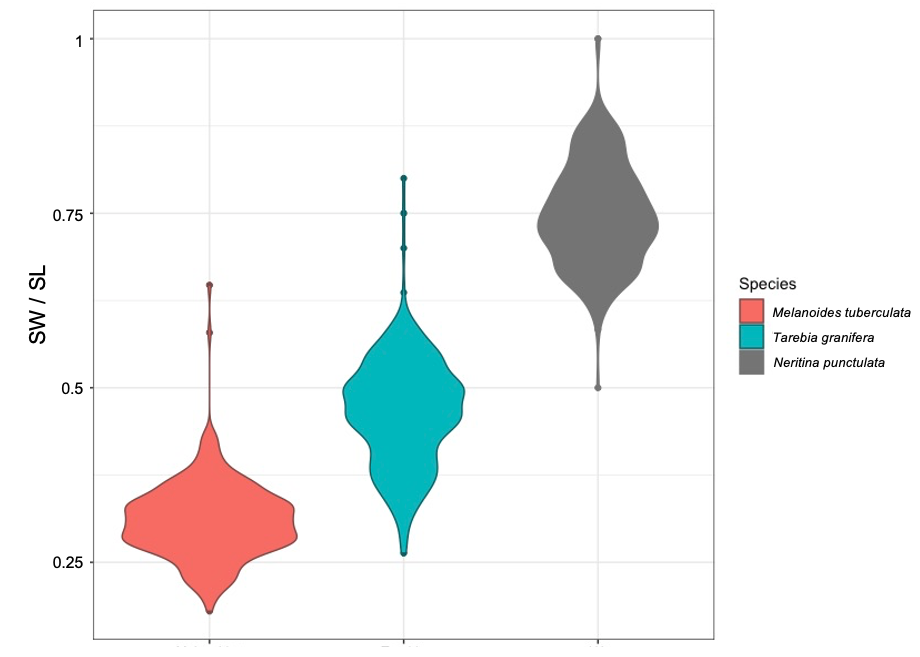


**Table S1.** Effect of total length (TL) of crayfish (*Cherax quadricarinatus*) on the main claw length (MC), considering sex (female and male) as a fixed effect. *t* and *P* are the results of the associated t-test and its p-value.

|  | **Estimate** | **Standard error** | ***t*** | ***p*** |
| --- | --- | --- | --- | --- |
| Intercept | -16.31 | 2.384 | -6.841 | < 0.001 |
| Total length | 0.468 | 0.024 | 19.568 | < 0.001 |
| Sex (Male) | 3.328 | 1.298 | 2.563 | 0.01 |

**Table S2A**. Morphometric measurements in male and female *Cherax quadricarinatus*. Mean value and range (in brackets) of total length (TL), carapace length (CL), main claw length (MC), width (CW), and abdomen width (Abd) – all in mm. Weight is in g. See Figure S4 for measurement representation.

|  | TL | CL | MC | CW | Abd | Weight |
| --- | --- | --- | --- | --- | --- | --- |
| Males | 123.6  (85 – 210) | 58.75  (30 – 110) | 44.8  (24 – 110) | 14.65  (7 – 34) | 23.59  (16 – 39) | 54.42  (15 – 188) |
| Females | 94.5  (51 – 137) | 43.81  (25 – 64) | 27.89  (13 – 43) | 7.24  (3 – 11) | 17.5  (10 – 27) | 21.34  (4 – 93) |

**Table S2B. Morphometric measurements in three snail species (*Melanoides tuberculata, Neritina punctulata* and *Tarebia granifera*). Mean value and range (in brackets) of shell length (SL), shell width (SW), aperture length (AL) and width (AW) – all in mm. Figure S7 show a shape representation of each species, based on a SW/SL ratio.**

|  | **SL** | **SW** | **AL** | **AW** |
| --- | --- | --- | --- | --- |
| ***Melanoides tuberculata*** | **13.7**  **(5 – 21)** | **4.3**  **(2 – 11)** | **4.2**  **(1 – 9)** | **3**  **(1 – 6)** |
| ***Neritina punctulata*** | **7.96**  **(3 – 12)** | **5.9**  **(2 – 9)** | **3.5**  **(1 – 6)** | **5.6**  **(2 – 9)** |
|  |  |  |  |  |
| ***Tarebia granifera*** | **14.5**  **(3 – 31)** | **6.7**  **(1 – 12)** | **6.5**  **(1.5 – 19)** | **4.3**  **(1 – 13)** |
